# Study of the physicochemical characteristics, antimicrobial activity, and in vitro multiplication of wild blackberry species from the Peruvian highlands

**DOI:** 10.1038/s41598-024-54058-0

**Published:** 2024-02-16

**Authors:** Yoiner K. Lapiz-Culqui, Jegnes Benjamín Meléndez-Mori, José Jesús Tejada-Alvarado, Denny Cortez, Eyner Huaman, Victor M. Núñez Zarantes, Manuel Oliva

**Affiliations:** 1 Instituto de Investigación Para el Desarrollo Sustentable de Ceja de Selva (INDES-CES), Universidad Nacional Toribio Rodríguez de Mendoza (UNTRM), 01001 Chachapoyas, Peru; 2 Facultad de Ciencias Agronómicas, Universidad de Chile, 11315 Santa Rosa, La Pintana, Santiago Chile; 3 Estación Experimental Agraria Amazonas, Dirección de Recursos Genéticos y Biotecnología (DRGB), Instituto Nacional de Innovación Agraria (INIA), Ex Aeropuerto, Fundo San Juan, 01001 Chachapoyas, Amazonas Peru; 4 Centro de Investigación Tibaitatá, Corporación Colombiana de Investigación Agropecuaria, AGROSAVIA, Mosquera, Colombia

**Keywords:** Antimicrobial, Antioxidant, Blackberry, Micropropagation, Biotechnology, Plant sciences

## Abstract

The Peruvian Andes are the natural habitat of several wild blackberry species that are little known and exploited due to the lack of technological and scientific development to support their agricultural potential. In this context, a study was conducted to understand the physicochemical composition, bioactive compounds, antimicrobial activity, and in vitro multiplication of four wild blackberry (*Rubus* sp.) species from the northern Peruvian highlands. The results indicate that fruits of *R. floribundus* presented the highest content of total soluble solids (9.58 ± 1.83°Brix) and titratable acidity (1.88 ± 0.07% citric acid). The fruits of *R. weberbaueri* recorded the highest total phenolic content (415.06 ± 8.69 mg GAE/100 g Ff). The antioxidant capacity determined by the DPPH assay varied significantly among species, with the highest value found in fruits of *R. andicola* (50.27 ± 0.11 mg TE/100 g Ff). The fruit extracts of *R. weberbaueri* and *R. andicola* showed better antimicrobial activity, with *Staphylococcus aureus* being the most sensitive bacterium. In the in vitro multiplication phase, the results show that BAP (6-Benzylaminopurine) has a significant effect at a dose of 1.5 mg l^−1^ on shoot number, leaf number, and shoot length. The results may help in the management of genetic resources.

## Introduction

Blackberry is one of the most widespread berries of the genus *Rubus* (Rosaceae), with species (wild and cultivated) widely distributed throughout the world. Today, the popularity of *Rubus* species has increased due to the high levels of anthocyanins and phenolic compounds in their fruits^1–3^; bioactive compounds with antioxidant and anticarcinogenic activity^4,5^.

Numerous wild species, many of which have the potential to be exploited in agriculture, can be found thriving in the high Andean lands of Peru^6^. These species are widely distributed throughout the territory due to the presence of ecological floors and climates conducive to their development. However, the fruits of the agrobiodiversity of the *Rubus* genus are little known and exploited due to the lack of knowledge of their physicochemical characteristics and the technological limitations that promote their domestication, valorization, and protection^7^.

According to a recent study, the genus *Rubus* contains the most diverse group of berries in the Andes in northern Peru (Amazonas region)^8^. These wild species can be distinguished by distinctive morphological characteristics, and their fruits can be consumed fresh or used for making liquor, ice cream, yogurt, or jam. They may have promising characteristics given that wild species have been observed to frequently include more phenols than domesticated species^9,10^. In addition, recent research has shown that a large portion of the fruits of this genus contain compounds that can inhibit or eliminate the development of pathogenic microorganisms^11–14^. However, it is common that the composition and healthful effects of bioactive substances from wild species are not thoroughly known^13,15^.

Wild blackberries have significant potential in the global market, but efforts to improve their propagation systems and promote the agricultural development of new *Rubus* species have been lacking^16^. Tissue culture is a practical alternative to encourage the cultivation of wild fruits with agricultural potential. This biotechnological tool allows obtaining numerous, healthy, and uniform offspring, while ensuring the transfer of the genetic potential of the parent^17,18^. However, factors such as species, explant source and growth regulator concentration have been shown to significantly influence in vitro culture^19,20^.

Therefore, it is important to develop studies that seek to decipher the specific composition of wild blackberries, their health benefits, as well as to optimize the use of growth regulators at each stage of micropropagation. In this context, the objective of the present study was to compare the physicochemical composition, the antioxidant capacity, the antimicrobial activity of four wild blackberry species from the northern Peruvian highlands, and to evaluate the in vitro response during multiplication under the influence of different concentrations of 6-benzylaminopurine (BAP). Thus, the results could provide important information to demonstrate the benefits of consuming this fruit and to assist in the management of genetic resources.

## Materials and methods

The study was carried out at the Laboratory of Plant Physiology and Biotechnology of the National University Toribio Rodríguez de Mendoza of Amazonas. First, vigorous plants of four wild *Rubus* species (*R. andicola*, *R. adenothallus*, *R. weberbaueri*, and *R. floribundus*) located in the districts of Granada and Molinopampa in northern Peru were selected, at an altitude between 2150 and 3250 m asl (Table 1; Table S1). These species are distinguished by their bushy form, with white, pink, and greenish flowers, and branches with thorns. The plants found grew naturally in acid soils, in Andean cloud forest ecosystems with a very humid climate, with temperatures ranging from 7 to 25 °C and an average annual accumulated rainfall of 1500 mm.

**Table 1:** Geographical location of wild species of *Rubus*.

Species	Geographical coordinates		Elevation
	Latitude South	Longitude West	
*R. weberbaueri*	6° 08′ 59.48″	77° 40′ 16.09''	3251
*R. floribundus*	6° 20′ 15.31″	77° 31′ 06.41''	2156
*R. adenothallus*	6° 06′ 07.01″	77° 38′ 28.79''	2822
*R. andicola*	6° 20′ 23.72″	77° 31′ 00.27″	2188

### Ethics statement

The experimental protocol was approved by the Institutional Committee on Research Ethics of the National University Toribio Rodríguez de Mendoza of Amazonas. The plant collection and use were in accordance with all the relevant guidelines. The identification of the species was reported by Tineo et al.^8^ in the research “Exploring the diversity of andean berries from northern Peru based on molecular analyses”. The samples were deposited in the KUELAP herbarium of the National University Toribio Rodríguez de Mendoza of Amazonas, with registration code KUELAP-254, KUELAP-255, KUELAP-256, and KUELAP-257.

### Evaluation of physicochemical characteristics, and antioxidant capacity of fruit

**Figure 1:**
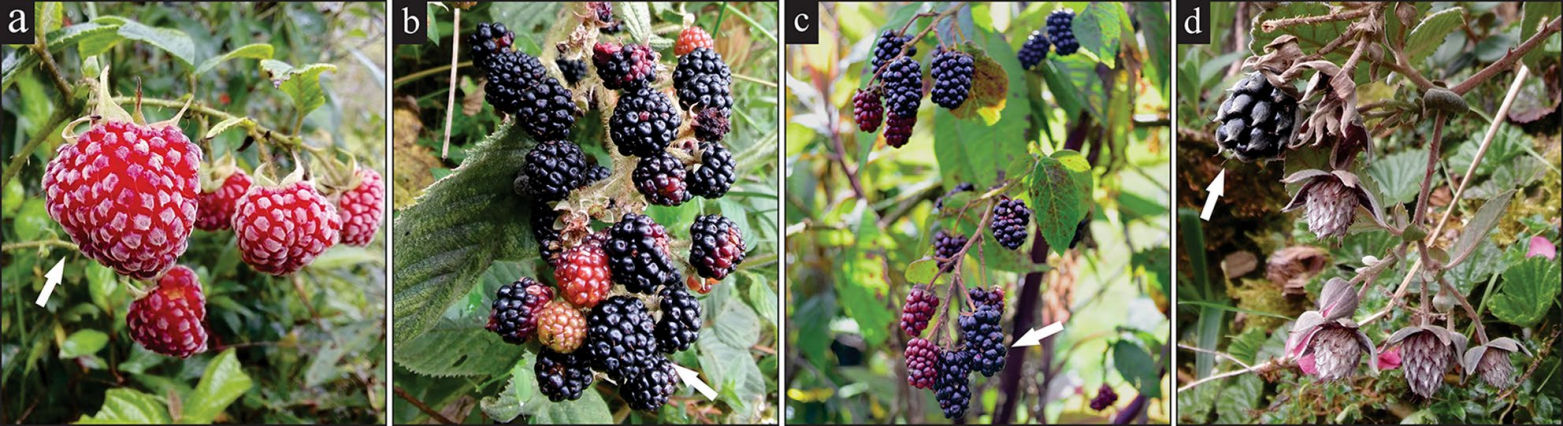
Wild blackberry fruits of (**a**) *R. andicola*, (**b**) *R. floribundum*, (**c**) *R. andenothallus*, and (**d**) *R. webebaueri*. The arrow indicates the maturity stage of fruits collected for analysis.

Fruits were harvested when they were completely commercially ripe, undamaged, and firm to the touch (Fig. 1). The longitudinal and transverse diameter was recorded with a digital vernier and the fresh weight with an analytical balance. In addition, firmness was measured with a digital penetrometer equipped with a 4 mm conical tip.

A pocket PAL-1 digital refractometer (Atago, Japan) was used to measure the total soluble solids content (°Brix). The pH of the pulp extract was determined with a HANNA HI2211 potentiometer (Hanna, USA). Titratable acidity was determined by potentiometric titration using a 0.1 N NaOH solution to pH 8.2 and the results were expressed as % citric acid.

To determine total phenolic compounds, the Folin-Ciocalteu method was used following the methodology of Fascella et al.^21^, with some modifications. Five g of freeze-dried fruit were combined with 50 mL of methanol/water (80:20, v /v) and then the mixture was centrifuged for 16 h at 400 rpm. Subsequently, a second centrifugation was completed at 5000 rpm for 15 min. Then, 1000 μL of supernatant and 2500 μL of Folin-Ciocalteu 2N were added into amber tubes. After 5 min, 2000 μL of 10% Na_2_CO_3_ was added, and the mixture was allowed to stand at room temperature for 2 h in the dark. Finally, the absorbance of the sample was measured at 725 nm on a Genesys 180 UV/Vis spectrophotometer (Thermo Scientific™, Madison, WI, USA). The calibration curve was created with dilutions of gallic acid equivalent (GAE) (0–180 mg L^−1^), leading to the equation: Y = 150.5x + 5.2; R^2^ = 0.993. Results were expressed as mg GAE/g Ff (freeze-dried fruit).

Antioxidant activity was determined by the DPPH method following the methodology of Albert et al.^22^, with some modifications. In an amber flask, 2.4 mg of DPPH (2,2-diphenyl-1- picrylhydrazyl) reagent was dissolved in 100 mL of methanol. The reagent was diluted with methanol to an absorbance of 0.70 ± 0.02 at a wavelength of 515 nm and left without light until use. In tubes containing 3.9 mL of DPPH solution, we added 100 μL of hydromethanolic extract. Subsequently, it was centrifuged for 1 min at 1800 rpm and allowed to stand in the dark for 30 min. The absorbance of the sample was measured at 515 nm on a Genesys 180 UV/Vis spectrophotometer (Thermo Scientific™, Madison, WI, USA). The results were expressed as mg Trolox equivalent (TE)/g Ff. The regression equation was y = 199.29x + 0.3958 (R^2^ = 0.996). The percentage inhibition activity was calculated as: E % = [(A0 − A1)/A0] × 100, where: A0 = the initial absorbance, and A1 = the absorbance in the presence of the extract.

### Evaluation of the antimicrobial activity of blackberry extract

The antimicrobial activity of the hydromethanolic extract (mixture of 2 g of fruit with 2.5 mL of methanol/water (80:20, v/v)) of blackberry was evaluated against a Gram-positive bacterium (*Staphylococcus aureus* ATCC29213) and a Gram-negative bacterium (*Escherichia coli* ATCC25922) donated by the Regional Health Directorate—Amazonas, Peru.

To evaluate the antimicrobial activity of blackberry extract, the methodology described by Chen et al.^23^ was followed, with some modifications. Bacterial strains were inoculated in Mueller–Hinton (MH) broth and incubated for 24 h at 37 °C. Each strain inoculum was suspended in sterile 0.85% saline and the bacterial population was adjusted spectrophotometrically OD_600_ to the 0.5 McFarland (used to standardize the approximate number of bacteria in a liquid suspension by comparing the turbidity of the suspension with that of the McFarland standard) standards (1.5 × 10^8^ CFU/mL).

The antimicrobial activity of the extracts was evaluated by detection of diameter of inhibition zone (DIZ). Bacterial suspensions (1.5 × 10^8^ CFU/mL) were seeded uniformly on the culture medium and a sterile paper disk (6 mm) impregnated with the extract (8 μL) of blackberries was placed. It was incubated for 24 h at 37 °C. Sterile distilled water (8 μL) was used as a negative control and ampicillin (50 μg/disc) and oxacillin (50 μg/disc) as a positive control for *E. coli* and *S. aureus*, respectively.

### Evaluation of in vitro multiplication

Actively growing tillers were collected and set up in 4" × 8" polyethylene bags containing a peat and rice husk-based substrate in a 3:1 ratio with a pH of 5.7. Plants were grown in a nursery for two months at a temperature of 20 ± 2 °C and relative humidity > 70%.

For in vitro establishment, sterile explants (2 cm segments with an axillary bud) were grown on MS culture medium^24^ supplemented with 100 mg myo-inositol, 30 g sucrose, 150 mg ascorbic acid and 6.5 g agar. The pH of the medium was adjusted to 5.8 and autoclaved at 120 °C for 20 min. Cultures were grown for 30 days in a growth room at 20 ± 2 °C and a 16-h photoperiod.

For the multiplication phase, explants adapted to the establishment medium were selected. These were extracted and cut into 1 cm fragments with an axillary bud. The explants were introduced into establishment medium supplemented with different concentrations of 6-benzylaminopurine (1.0, 1.5 and 2.0 mg l^−1^) and a control treatment. The pH of the medium was adjusted to 5.8 and autoclaved at 120 °C for 20 min. The explants were grown at a temperature of 20 ± 2 °C and a photoperiod of 16 h light with 1.5 k lux light intensity. At 42 days, the number of shoots, number of leaves and shoot height were evaluated. In addition, the percentage of explant loss was determined.

### Experimental design and statistical analysis

The trials were conducted under a completely randomized design. A one-way analysis of variance was performed for fruit quality and antimicrobial activity parameters, while a two-way analysis of variance was performed for in vitro multiplication parameters. Each treatment consisted of 10 replicates. Means were compared by Tukey test at 5% probability of error. The analysis was performed with the statistical package InfoStad version 2020p.

## Results 

### Evaluation of physicochemical characteristics, and antioxidant capacity of fruit

Morphometric attributes of wild blackberry fruits showed significant variation among species (Table 2). The fruits of *R. andicola* were characterized by greater longitudinal (27.27 ± 5.16 mm) and transverse (21.48 ± 2.90 mm) growth. In addition, it is evident that the fruits of the species (*R. andicola*) had a higher weight (7.56 ± 1.27 g) due to the growth achieved. All the variables contained in Table 2 show that *R. floribundus* presents the lowest data.

**Table 2:** Morphometric fruit quality of four wild blackberry species.

Species	Longitudinal diameter (mm)	Transverse diameter (mm)	Weight (g)	Firmness (N)
*R. andicola*	27.27 ± 5.16 a	21.48 ± 2.90 a	7.56 ± 1.27 a	4.29 ± 1.01 bc
*R. adenothallus*	25.11 ± 1.34 ab	16.43 ± 1.15 b	4.12 ± 0.29 b	5.09 ± 0.78 b
*R. floribundus*	11.57 ± 0.39 c	11.43 ± 0.23 c	0.92 ± 0.11 d	2.86 ± 0.79 c
*R. weberbaueri*	20.67 ± 1.60 b	14.92 ± 0.28 b	2.38 ± 0.15 c	6.22 ± 1.05 a

Means within a column followed by the same letter are not significantly different according to Tukey’s test at *P* ≤ 0.05.

In contrast to the morphometric characteristics, the fruits of *R. floribundus* showed a higher content of total soluble solids with 9.58 ± 1.83°Brix. In titratable acidity, measured as percentage of citric acid, it is also observed that *R. floribundus* stands out over the other species with 1.88 ± 0.07% (Table 3). Regarding the content of total phenols, the fruits of wild blackberries showed significant variation among species (Table 4), with *R. weberbaueri* presenting the highest concentrations with 415.06 ± 8.69 mg GAE/g Ff. On the other hand, it was observed that the antioxidant capacity (determined by DPPH radical binding activity) of each blackberry species was significantly different, having the highest value in fruits of *R. andicola* (50.27 ± 0.11 mg TE/g Ff), followed by *R. floribundus* (49.59 ± 0.40 mg TE/g Ff). Overall, the data obtained showed that wild blackberries had had strong DPPH free radical scavenging effects (> 95% inhibition).

**Table 3:** Comparison of total soluble solids (°Brix), pH and titratable acidity contents of fruits of four wild blackberry species.

Species	Brix	pH	Titratable acidity (% citric acid)
*R. andicola*	6.96 ± 0.09 b	2.87 ± 0.02 d	1.60 ± 0.11 b
*R. adenothallus*	6.36 ± 1.09 b	3.13 ± 0.01 b	1.30 ± 0.04 c
*R. floribundus*	9.58 ± 1.83 a	2.94 ± 0.03 c	1.88 ± 0.07 a
*R. weberbaueri*	5.28 ± 0.11 b	3.32 ± 0.01 a	1.17 ± 0.04 c

Means within a column followed by the same letter are not significantly different according to Tukey’s test at *P* ≤ 0.05.

**Table 4:** Total phenol content and antioxidant capacity of fruit extracts from four wild blackberry species.

Species	Total phenolic content (mg GAE/g Ff)	E% (DPPH Assay)	Antioxidant capacity (mg TE/g Ff)
*R. andicola*	119.38 ± 3.30 c	98.79 ± 0.91 a	50.27 ± 0.11 a
*R. adenothallus*	179.53 ± 16.68 b	96.76 ± 0.43 b	48.77 ± 0.22 b
*R. floribundus*	230.15 ± 6.06 b	98.10 ± 0.25 a	49.59 ± 0.40 a
*R. weberbaueri*	415.06 ± 8.69 a	95.98 ± 0.97 b	48.19 ± 0.59 b

Means within a column followed by the same letter are not significantly different according to Tukey’s test at *P* ≤ 0.05.

### Evaluation of antimicrobial activity of blackberry extract.

**Figure 2:**
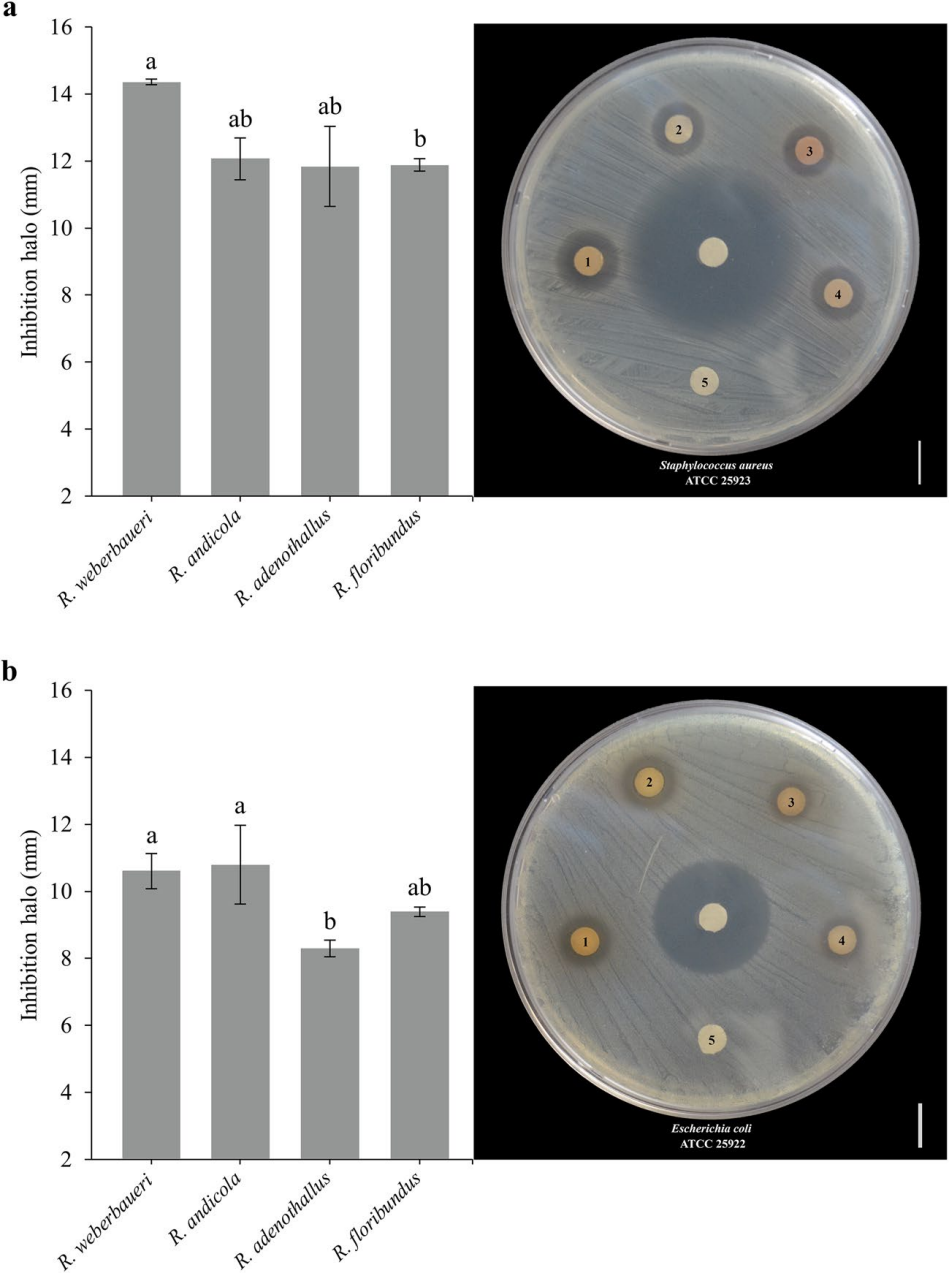
Diameter of antibacterial inhibition of wild blackberry extract against (**a**) *Staphylococcus aureus* and (**b**) *Escherichia coli* bacteria. The discs are labeled 1 for *R. webebaueri*, 2 for *R. andicula*, 3 for *R. andenothallus*, 4 for *R. floribundum*, and 5 for distilled water. The center disk includes the antibiotic ampicillin and oxacillin.

The determinations of DIZ (Fig. 1) demonstrate the high antimicrobial potential of blackberry extracts against *Staphylococcus aureus* (Fig. 2a) and *Escherichia coli* (Fig. 2b). *R. weberbaueri* is particularly notable for showing a wider inhibition halo against *S. aureus* (Disc 1 = 14 ± 1.26 mm), which places it above the other species that showed an inhibition halo of 11.5–12 mm. On the other hand, it was observed that the extracts of *R. weberbaueri* and *R. andicola* were the ones that neutralized *E. coli* to the greatest extent. With *R. andenothallus* (Disc 3 = 8 ± 0.23 mm), the minimum inhibition halo was reached.

### Evaluation of in vitro multiplication

**Figure 3:**
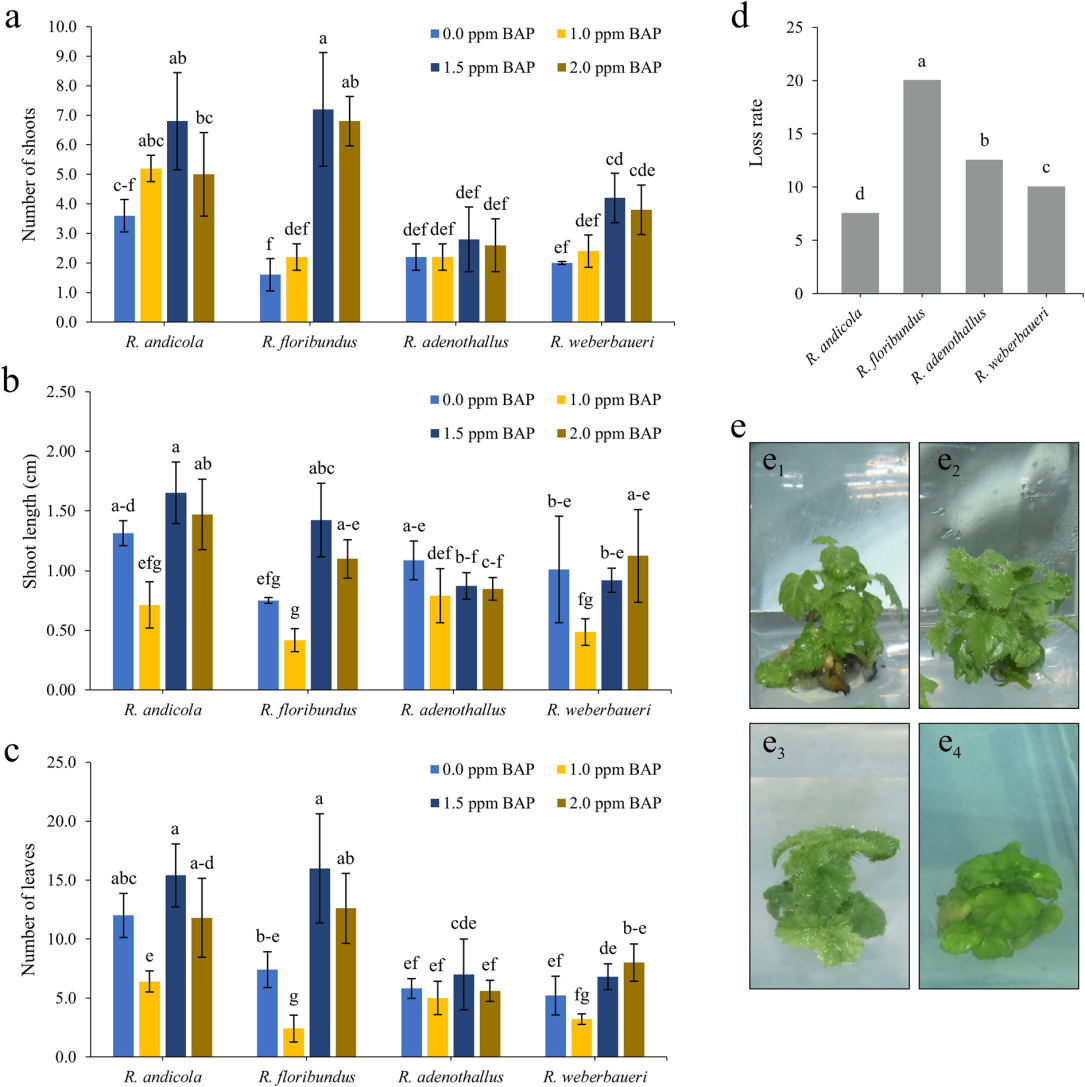
Effect of different concentrations of 6-benzylaminopurine on in vitro multiplication of four wild blackberry species. Panels are (**a**) number of shoots, (**b**) shoot height, (**c**) number of leaves, (**d**) loss rate and (**e**) in vitro plant regeneration. (**e**_**1**_) *R. andicola*, (**e**_**2**_) *R. floribundus*, (**e**_**3**_) *R. adenothallus*, and (**e**_**4**_) *R. weberbaueri*.

The results show that, except for *R. adenothallus*, all species developed on average more than 3 shoots per explant when grown on medium supplemented with 1.5 and 2 mg l^−1^ BAP. Among all species, *R. floribundus* produced the highest number of shoots (7.2 ± 1.9) when grown under the influence of 1.5 mg l^−1^ BAP (Fig. 3a–e1). On the other hand, it is observed that when the medium with 1.5 and 2.0 mg l^−1^ of BAP was used, *R. andicola* shoots reached a growth of more than 1.2 cm (Fig. 3b). Explants of *R. floribundus* and *R. weberbaueri*, which were grown on medium with 1.0 mg l^−1^ BAP, had significantly lower shoot height (0.42 and 0.49 cm, respectively).

Regarding the number of leaves, *R. andicola* and *R. floribundus* developed on average more than 8 leaves when grown on medium supplemented with 1.5 and 2.0 mg l^−1^ of BAP. These doses improved the average number of leaves obtained by *R. adenothallus* and *R. weberbaueri*, but there were no significant differences under any treatment (Fig. 3c). Finally, explant loss varied between 7.5 and 20%, with a minimum in *R. andicola* and a maximum in *R. floribundus* (Fig. 3d). In general terms, the four species showed a good survival rate, although marked differences were observed in their development, with *R. andicola* and *R. floribundus* being the species with the best response during the multiplication phase (Fig. 3e).

## Discussion

Regardless of the species, the pH and acidity values found in the fruits evaluated in this study were as expected, since ripe fruits of Rubus species have pH values ranging from 2.65 to 3.20 and titratable acidity values of 0.29 and 2.3%^25^. Regarding total soluble solids, Rubus fruits generally register values around 10°Brix^26–28^. Thus, it can be said that the results indicate that a species contains a % of sugar that is within the range of expected values. It should be noted that although the development of sweetness is crucial, acidity also influences the taste of the fruit, which provides a proper balance of sugar and acid for a pleasant taste^29^.

Raspberries and blackberries are known to be good sources of phenolic compounds^30^, which are strongly related to antioxidant capacity^31^. In addition, there is evidence to support that wild berries have higher levels of phenolic content and antioxidant capacity compared to domesticated and genetically derived crops^32^. In this study, the total phenolic contents of the species studied ranged from 119.38 to 415.06 mg GAE/g Ff. These results were higher than those obtained by other researchers for fruit extracts of *R. fruticosus* (31.05 mg GAE/g dw)^33^ and five blackberry cultivars produced in Brazil (8.23–14.98 mg GAE/g fw)^34^, and for example were in the range of 226.58–392.9 mg GAE/g reported for root extract of *R. hyrcanus*^35^. However, it is important to understand that a variety of factors, including species, climate, soil, ripening phase, location of the fruit on the plant, sun exposure, maturity, post-harvest handling, and different extraction methods, can influence the concentration of these compounds^36^. All these factors may help explain the differences in phenolic concentrations between the different species examined. On the other hand, the DPPH method used to evaluate the antioxidant capacity of the extracts shows that the antioxidant capacity values of the blackberry species examined ranged from 48.19 to 50.27 mg TE/g Ff. Other researchers obtained lower values in *R. coreanus* extracts (16.25 mg TE/g dFW)^23^; however, both investigations demonstrated a high capacity to scavenge DPPH radicals (> 90%). In addition, it was observed that the DPPH radical inhibition efficiency of blackberries in this study was higher than that reported for blackberries of Brazos, Tupy, Arapaho, Choctaw, and Guarani cultivars, which remained in the range of 50–75%^37^.

Within modern medicine, trends are aimed at overcoming mortality and morbidity problems as a result of the emergence of a resistance of several pathogenic microorganisms to a variety of drugs^13,38^. In this context, species of the genus *Rubus* have been reported to be high in flavonoids, tannins, carbohydrates, ascorbic acid, organic acids, and volatile oils^39,40^. Of these, phenolic compounds exhibit antibacterial mechanisms such as the ability to inhibit nucleic acid synthesis, cytoplasmic membrane functions, biofilm binding and formation, and alter membrane permeability^41^. These compounds have been shown to be effective against *Gram-negative bacteria* such as *E. coli* and *Pseudomonas aeruginosa*^42^. Our findings showed that the DIZ values of the blackberry extracts were in the range of 8.29–14.33 mm for the two tested bacteria, especially highlighting the *R. weberbaueri* and *R. andicola* extracts. These results are in the range of DIZ values reported for *R. coreanus* extract against eleven different foodborne pathogens^23^. In general, it is observed that Gram-negative and Gram-positive bacteria have different sensitivities to antibacterial substances. This may be due to the fact that the outer membrane of the former is composed of hydrophobic lipopolysaccharides, which give them greater resistance^43,44^.

It has been discovered that berries have a high content of phytochemicals (dependent on genetic and environmental factors) that allows them to combat different microorganisms to a greater or lesser extent^45^. The microbicidal activity of blackberry extracts has been widely reported on a wide range of large-positive and large-negative bacteria. For example, extracts of *R. idaeus*, *R. moluccanus* L*., R. fraxinifolius* Poir., *R. alpestris* Blume*, R.* rosaefolius, and *R. chingii,* demonstrated high efficacy agains*t Streptococcus typhi*, *Streptococcus pneumoniae*, *Staphylococcus aureus, Streptococcus epidermidis, Moraxella catarrhalis, Haemophilus influenzae, Helicobacter pylori, Klebsiella pneumoniae, Enterococcus faecalis*, *Bacillus subtilis, E. coli, Salmonella enteritidis, Aeromona hydrophila*, *Pseudomonas aeruginosa,* and *Pseudomonas fluorescens*^11,14,46–48^. On the other hand, it has been reported that the extracts of these fruits do not affect lactic acid bacteria, used in the fermentation processes of foods^48,49^. This demonstrates that wild fruits are an important source of potentially healthy phytochemical compounds.

In in vitro multiplication, the use of cytokinins to activate cell division and induce the development of new shoots is frequent^50^. For example, in the in vitro multiplication of blackberries, the use of BAP has been shown to have significant effects; however, the effect varies depending on the cultivar or genotype to be propagated^1,51^. In the present study, variation in the in vitro response was not only associated with the source of the explant (species), but also with the concentration of cytokinin (BAP); suggesting an interaction of genotype with the levels of BAP used.

According to the results, it can be mentioned that the variables number and length of shoots were favored by the presence of 1.5 mg l^−1^ of BAP in the culture medium, but, after this level, they showed a decreasing trend. A similar effect was described by Kefayeti et al.^52^ and Schiehl et al.^53^, as they point out that multiplication rates and shoot growth decreased when the culture medium exceeded a certain concentration of BAP. The difference in response to the optimal BAP concentration for in vitro multiplication is likely due to variation between genotypes. In general, determining the appropriate cytokinin concentration decreases the likelihood of toxic effects, while maintaining good multiplication rates and flare-up characteristics^54^. In addition, the results allow us to generate a baseline for future studies focused on determining the optimization of culture media by genotype or by group of genotypes with the addition of other growth regulators such as gibberellic acid that stimulates shoot elongation and improves leaf formation.

Regarding leaf formation, the results of the present study were superior to the report of Millones^6^, who when using 0.1 mg l^−1^ of BAP recorded up to 5.5 leaves per explant in a wild blackberry accession. On the contrary, Cancino-Escalante et al.^16^ reported that the use of 2 mg l^−1^ of BAP + 1 mg l^−1^ of AG_3_ achieved an average of 14.54 leaves/explant, which is higher than the report of this study. Variations between the above studies may be related to the use of different concentrations of BAP. However, several reports indicate that the use of BAP does not have significant effects on the number of leaves formed^55–57^, which suggests that they are more closely linked to the response variable of the different genotypes.

The rate of loss of explants reveals that some species are more susceptible than others. These variations, as well as morphometric characteristics, are influenced by genetic factors that determine the sensitivity of plant material, reflecting a variable in vitro growth and morphogenesis response with the genotype^58^. In the study, explant survival was affected by explant oxidative processes. This process is since explants usually excrete phenolic compounds that manage to oxidize and lead to the generation of radicals that are highly toxic to plant tissues^59^. Therefore, future studies should consider the addition of antioxidants to the culture medium. BAP generally induces budding, but can affect elongation, leaf production, and rooting, depending on the genotype. In this study, explants grown in BAP medium showed callus formation but not roots, evidencing a possible negative effect of this cytokinin on root development.

## Conclusions

The results obtained in our study show that the fruits of wild blackberries from the Peruvian highlands have high antioxidant capacity and antimicrobial activity against *E. coli* and *S. aureus*, highlighting their potential to produce functional foods. Evaluations showed that the fruits of *R. floribundus* presented high content of total soluble solids and titratable acidity. The fruits of *R. weberbaueri* had the highest total phenolic content. The antioxidant capacity (DPPH assay) of the fruits of the four species varied significantly, but in all cases, they showed the capacity to inhibit more than 95% of the DPPH radicals. The fruit extracts of *R. weberbaueri* and *R. andicola* showed higher levels of antimicrobial activity, with *S. aureus* as the most susceptible bacterium. Results from the in vitro multiplication phase showed that BAP had a significant impact on shoot number, leaf number and shoot length at a dose of 1.5 mg L^−1^. In addition, it was observed that *R. andicola* and *R. floribundus* were the species that showed the most effective response at this stage.

### Supplementary Information

Supplementary Table S1.

## Data Availability

The datasets generated during and/or analyzed during the current study are available from the corresponding author on reasonable request.
