# Supplementary material for: Study of the physicochemical characteristics, antimicrobial activity, and in vitro multiplication of wild blackberry species from the Peruvian highlands
Source: Sci Rep. 2024 Feb 16;14:3863. doi: 10.1038/s41598-024-54058-0 (PMC10873364; doi:10.1038/s41598-024-54058-0)
Supplement: Supplementary file 1 — Supplementary Table S1. [file 41598_2024_54058_MOESM1_ESM.docx]

Supporting Information

**Study of the physicochemical characteristics, antimicrobial activity, and in vitro multiplication of wild blackberry species from the Peruvian highlands**

Yoiner K. Lapiz-Culqui^1^, **Jegnes Benjamín Meléndez-Mori^1^*,** José Jesús Tejada-Alvarado^1,3^, Denny Cortez^1^, Eyner Huaman^1,2^, Victor M. Núñez Zarantes^4^, Manuel Oliva^1^

^1^Instituto de Investigación para el Desarrollo Sustentable de Ceja de Selva (INDES-CES), Universidad Nacional Toribio Rodríguez de Mendoza (UNTRM), Chachapoyas 01001, Peru.

^2^Universidad de Chile, Facultad de Ciencias Agronómicas, Santa Rosa 11315, La Pintana, Santiago, Chile.

^3^Estación Experimental Agraria Amazonas, Dirección de Recursos Genéticos y Biotecnología (DRGB), Instituto Nacional de Innovación Agraria (INIA). Ex aeropuerto, Fundo San Juan, Chachapoyas, 01001, Amazonas, Peru.

^4^Corporación Colombiana de Investigación Agropecuaria, AGROSAVIA, Centro de Investigación Tibaitatá, Mosquera, Colombia.

***Corresponding Author Email:** [**jbenjamin@indes-ces.edu.pe**](mailto:jbenjamin@indes-ces.edu.pe) **(Jegnes Benjamín Meléndez-Mori)**

**Table S1:** Chemical and physical characteristics of the soil in the plant collection area of four wild blackberry species.

| **Properties** | ***R. adenothallus*** | ***R. andicola*** | ***R. floribundus*** | ***R. weberbaueri*** |
| --- | --- | --- | --- | --- |
| PH | 5.41 | 4.93 | 5.99 | 4.23 |
| EC (dS/m) | 0.15 | 0.40 | 0.31 | 0.15 |
| Organic matter (%) | 6.90 | 6.86 | 7.62 | 7.38 |
| CEC (meq/100g) | 12.80 | 12.00 | 19.20 | 6.40 |
| Total organic C (%) | 4.00 | 3.98 | 4.42 | 4.28 |
| Total N (%) | 0.34 | 0.34 | 0.38 | 0.39 |
| C/N Ratio | 11.76 | 11.71 | 11.63 | 10.97 |
| Available P (mg/kg) | 22.10 | 50.07 | 22.51 | 92.02 |
| Exchangeable K (meq/100 g) | 1.03 | 1.22 | 0.49 | 0.40 |
| Exchangeable Ca (meq/100 g) | 7.72 | 5.11 | 15.10 | 1.40 |
| Exchangeable Na (meq/100 g) | 0.10 | 0.12 | 0.10 | 0.09 |
| Exchangeable Mg (meq/100 g) | 2.11 | 4.95 | 1.62 | 0.57 |
| Textural class | Sandy loam | Sandy loam | Sandy loam | Sandy loam |
